# Regulation of UCP1 in the Browning of Epididymal Adipose Tissue by *β*3-Adrenergic Agonist: A Role for MicroRNAs

**DOI:** 10.1155/2014/530636

**Published:** 2014-12-17

**Authors:** Zongji Zheng, Xiaomeng Liu, Qianwei Zhao, Lei Zhang, Chenzhong Li, Yaoming Xue

**Affiliations:** ^1^Department of Endocrinology and Metabolism, Nanfang Hospital, Southern Medical University, Guangzhou 510150, China; ^2^Key Laboratory of Animal Ecology and Conservation Biology, Institute of Zoology, Chinese Academy of Sciences, Beijing 100101, China

## Abstract

*Background.* White adipose tissue browning may be a promising strategy to combat obesity. UCP1 is strongly induced in White adipose tissue with *β*3-adrenergic agonist treatment, but the causes of this increase have not been fully elucidated. This study aims to explore more miRNAs involved in the process of browning of visceral adipose tissue. *Methods.* Total of fourteen mice were randomly divided into control and study group. Study group mice were injected intraperitoneally with CL316243 once daily for seven days; meanwhile the control group were treated with 0.9% NaCl. After a 7-day period, the expression of genes involved in WAT browning and potential UCP1-targeting miRNAs in adipose tissues was analyzed by qPCR. *Results.* qPCR analysis revealed that UCP1, DIO2, CIDEA, and CPT1B in epididymal adipose tissue were overexpressed in CL316243 group. Furthermore, potential UCP1-targeting miR-9 and miR-338-3p in epididymal adipose tissue were significantly decreased in CL316243 group. *Conclusion.* This suggests that potential UCP1-targeting miR-9 and miR-338-3p may be involved in the browning of epididymal adipose tissue by regulating UCP1 gene expression. In this study, we demonstrated that this increase of UCP1 is due, at least in part, to the decreased expression of certain UCP1-targeting miRNAs in epididymal adipose tissue compared to control.

## 1. Introduction

With development of economy and changes in lifestyle, the epidemic of obesity and related metabolic syndrome is alarming, which has become one of the most important public health problems worldwide. Obesity is a well-known factor inducing diseases such as hypertension, coronary heart disease, and diabetes [1].

Obesity develops when energy intake exceeds the consumption [2, 3]. Dieting and exercise are generally considered to be the most effective treatments for obesity, yet long term persistence is difficult. Most antiobesity drugs have been withdrawn due to their adverse effects, such as psychiatric disorders and nonfatal myocardial infarction. In 2009, five independent researchers identified and characterized the presence of brown adipose tissue (BAT) in adult using 18F-FDG PET/CT [4–8], evoking attentions on using BAT to counter the spread of obesity due to its immense capacity to convert excess energy into heat. Increasing evidences indicated that BAT recruitment [9–13] and BAT transplantation [14, 15] may play an important role in decreasing body weight and improving whole-body energy metabolism.

Mammalian adipose tissue can be divided into white adipose tissue (WAT) and brown adipose tissue (BAT). WAT is characterized by a single, large lipid droplet and few mitochondria that stores excess energy in form of triglycerides, which also secretes a variety of cytokines to regulate energy metabolism [16]. WAT can be further divided into subcutaneous adipose tissue and visceral adipose tissue roughly. It is well established that visceral adipose tissue plays an important part in the pathophysiology of insulin resistance. Visceral adipose tissue makes obese individuals more prone to metabolic and cardiovascular diseases than fat distributing subcutaneously. BAT contains numerous small lipid droplets and a much higher number of mitochondria [17]. At the molecular level, uncoupling protein 1 (UCP1), which is uniquely expressed in BAT, is bound up with the heat production process [18]. UCP1 is localized on the inner mitochondrial membrane and uncouples the activity of the respiratory chain from ATP synthesis, thereby releasing energy as heat [17]. In humans, it was estimated that as little as 50 g of BAT could utilize up to 20% of basal caloric needs if maximally stimulated [19]. A large quantity of studies has demonstrated that *β*-adrenergic receptors participate in ageing pathophysiology [20]. Previous studies suggest that *β*3-adrenergic agonist induce UCP1 expression in WAT. Despite these evidences, the mechanisms that lie behind this increase are still unknown.

miRNAs are small noncoding RNAs of ~22 nucleotides in length that play a crucial part in posttranscriptional gene regulation [21]. miRNAs are involved in the pathogenesis of cardiovascular diseases and have become an intriguing target for therapeutic intervention [22–25]. In addition, miRNAs might serve a valuable diagnostic function for cardiovascular pathologies because miRNAs leak into circulating blood from injured cells [26]. miRNAs recently were confirmed as key regulators of metabolism [27] and induced transdifferentiation [28]. miRNAs have also recently been introduced as the key regulators of brite adipocyte development. Adrenergic stimulation inhibits expression of miR-133 (a muscle-enriched miRNA) to abolish posttranscriptional silencing of PRDM16. Inhibition of miR-133 increases the expression of PRDM16 and the mitochondrial activity [29]. MiR-155-deficient mice exhibit activated brown adipose tissue function and browning of white fat tissue [30]. MiR-196a induces functional brown adipogenesis in WAT through the suppression of Hoxc8 [31].

In vivo, the “brite/beige” cells are described as UCP1-positive islets within white fat depots following cold or *β*-adrenergic stimulation [18]. Meanwhile, thyroxine (T3) could also induce UCP1 expression in white adipocytes in our previous study [32]. However, whether more miRNAs are involved in the process of WAT browning especially epididymal adipose tissue (visceral adipose tissue) needs further study. In this study, we investigated the regulation and involvement of miRNAs in the browning of epididymal adipose tissue. The effects of CL316243 on the expressions of genes involved in WAT browning were detected and relevant regulatory miRNAs were analyzed using bioinformation software. Here, we sought to observe whether members of miRNA were involved in the process of browning of visceral adipose tissue and whether it could serve as a novel treatment for obesity.

## 2. Materials and Methods

### 2.1. Animal Model

For CL316243 treatment, fourteen six-week-old male C57BL/6J mice (purchased from Vital River Laboratory Animal Technology Co. Ltd.) were randomly divided into two groups, seven for each. One group was injected intraperitoneally (i.p.) once daily with 1 mg/kg CL316243 (Tocris Bioscience) in 0.9% NaCl for 7 days [33]; 0.9% NaCl was used in the control group instead of CL316243. The mice were maintained at 22 ± 2°C on a 12 h/12 h light cycle (8.00 a.m. to 8.00 p.m.) in Office of Laboratory Animal Welfare, certified animal facility, with free access to water and standard laboratory chow diet. Institutional Animal Care and Use Committee approved all experimental plans. Mice were killed by cervical dislocation. The interscapular brown adipose tissues, inguinal subcutaneous adipose tissues, and retroperitoneal epididymal adipose tissues were dissected out.

### 2.2. MicroRNA Target Site Predictions Algorithms

The miRNA target site predictions were performed using miRanda database (http://www.microrna.org/microrna/home.do).

### 2.3. Real-Time Quantitative PCR

Total RNA were isolated from brown adipose tissues (BAT), epididymal adipose tissues, and subcutaneous adipose tissues using TRIzol reagent (Invitrogen). RNA quality was assessed using NANODROP 2000 (Thermo Scientific). The cDNA was synthesized using random hexamers (Invitrogen) for subsequent real-time quantitative PCR analysis (ABI Prism VIIA7; Applied Biosystems Inc.). PCR products were detected using Sybr Green and normalized by cyclophilin A expression. Primers were designed using Primer Quest (Integrated DNA Technologies, Inc.). Primers for UCP1, PGC-1*α*, C/EBP*β*, PRDM16, PPAR*γ*2, CIDEA, DIO2, and CPT1B were listed in Table 1.

Small RNAs were extracted from brown adipose tissues, epididymal adipose tissues, and subcutaneous adipose tissues using the BiooPure RNA isolation kit (Bioo Scientific, Austen, TX, USA) according to the manufacturer's recommendations. RNA quality was assessed using NANODROP 2000 (Thermo Scientific). The cDNA was synthesized using miRcute miRNA first-strand cDNA synthesis kit (Qiagen) and PCR products were detected using miRcute miRNA qPCR detection kit (Qiagen) according to the manufacturer's recommendations for subsequent real-time quantitative PCR analysis (ABI Prism VIIA7; Applied Biosystems Inc.). The snRNA U6 was used as the internal control. Primers for miR-9, miR-338-3P, miR-let7g, and U6 were listed in Table 1.

The relative expressions among the different genes and miRNAs were determined using the 2^−ΔΔCT^ method.

### 2.4. Histochemistry

Adipose tissues were fixed overnight in 4% paraformaldehyde in PBS and embedded in paraffin. Samples were cut into 5 *μ*m sections, and hematoxylin-eosin staining was routinely performed.

### 2.5. Statistics

All data were presented as mean ± SEM. For all comparisons, Student's *t*-tests were performed using SPSS 21.0 software and *P* values less than 0.05 were considered to be significant on a two-tailed test.

## 3. Results

**Figure 1:**
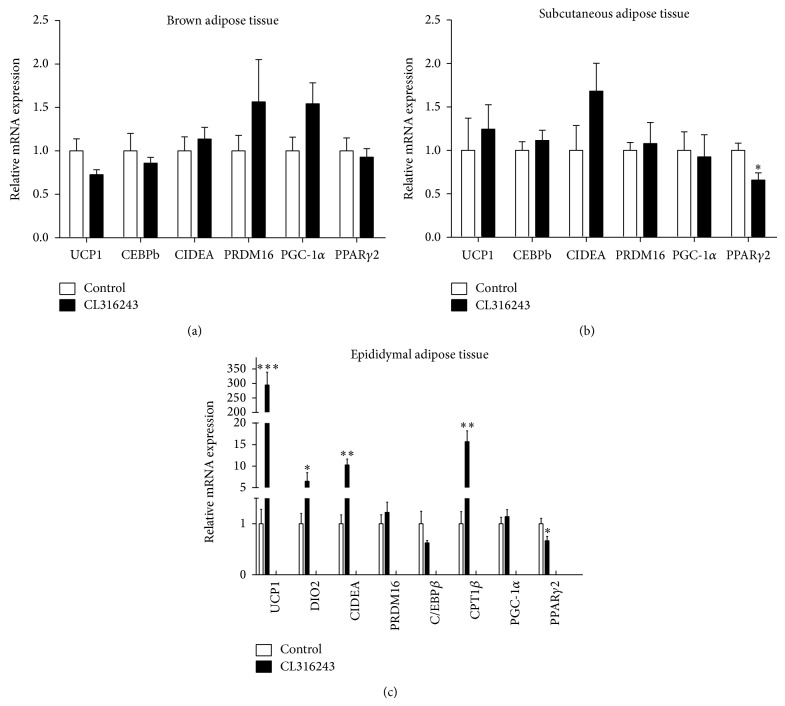
Analyses of mRNA expression of genes involved in WAT browning by qRT-PCR in brown adipose tissue, epididymal adipose tissue, and subcutaneous adipose tissue. (a) mRNA expression of genes involved in WAT browning in brown adipose tissue. (b) mRNA expression of genes involved in WAT browning in subcutaneous adipose tissue. (c) mRNA expression of genes involved in WAT browning in epididymal adipose tissue; *n* = 7 each. Values are mean ± SEM; ^*^ *P* < 0.05, ^**^ *P* < 0.01, and ^***^ *P* < 0.001.

*Effect of CL316243 on the Expression of Genes Involved in WAT Browning in the Adipose Tissues*. As a first step, we evaluated the expression of the genes involved in WAT browning in BAT with CL316243 treatment. We did not detect a significant change in the expression of genes involved in WAT browning such as UCP1, PGC-1*α*, C/EBP*β*, PRDM16, and CIDEA in BAT. As shown in Figure 1(a), compared with the control group, BAT specific genes UCP1, CIDEA and BAT differentiation genes PGC-1*α*, C/EBP*β*, and PRDM16 mRNA expression had no statistical differences in BAT of CL316243 treated group (*P* > 0.05).

Next, we investigated the effect of CL316243 treatment on the expression of the browning genes in subcutaneous adipose tissue. As shown in Figure 1(b), there were no significant changes in expression of the BAT specific genes UCP1, CIDEA and BAT differentiation genes PGC-1*α*, C/EBP*β*, and PRDM16 mRNA expression (*P* > 0.05).

**Figure 2:**
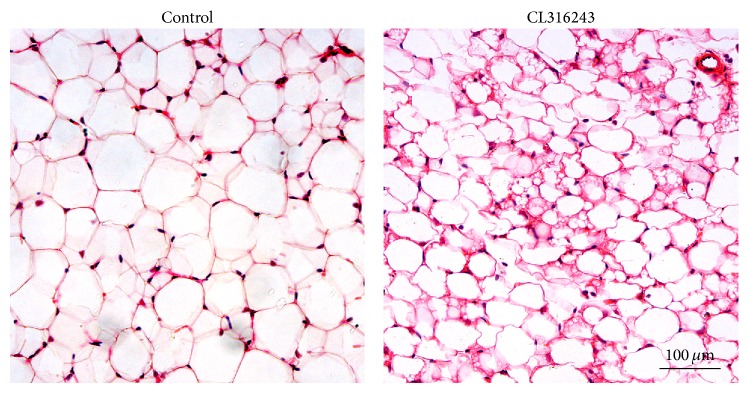
Photomicrographs of paraffin-embedded hematoxylin and eosin stained sections from epididymal adipose tissue.

Interestingly, as shown in Figure 1(c), BAT specific genes UCP1, CIDEA, DIO2, and fatty acid oxidation related gene CPT1B mRNA expression were significantly raised in epididymal adipose tissue with CL316243 treatment (*P* < 0.05). But the expression of other BAT differentiation related genes PGC-1*α*, C/EBP*β*, and PRDM16 mRNA had no statistical differences (*P* > 0.05). Histological examinations revealed typical unilocular cells with rich cytoplasmic staining and multilocular lipid droplets in CL316243 treatment group in epididymal adipose tissue (Figure 2).

**Figure 3:**
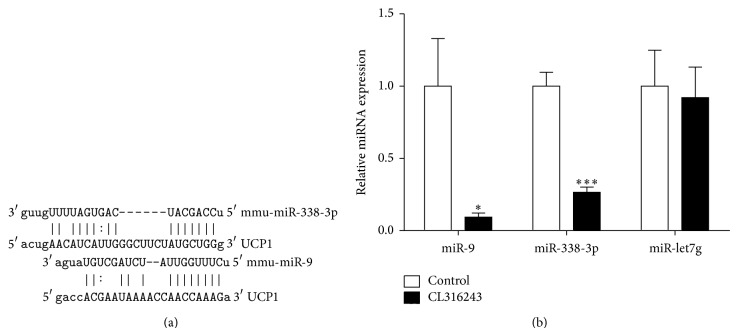
Analyses of mRNA expression of potential UCP1-targeting by qPCR in epididymal adipose tissue. (a) miRanda database predicts the binding site of UCP1 3′UTR region of miR-9 and miR-338-3p. (b) mRNA expression of miR-9, miR-338-3p, and miR-let7g mRNA in epididymal adipose tissue (*n* = 7 each). Values are mean ± SEM; ^*^ *P* < 0.05 and ^***^ *P* < 0.001.

*In Silico miRNAs Target Site Predictions*. It is well known that UCP1 is key to the brown fat adaptive thermogenesis. What is noteworthy is the fact that UCP1 mRNA expression was more significantly raised in the epididymal adipose tissue with CL316243 treatment than control group. We used the miRanda prediction algorithm to identify putative miRNAs that could regulate UCP1 genes. A panel of 2 miRNAs, namely, miR-9 and miR-338-3p, was selected for its putative ability to target the 3′-UTRs of UCP1 mRNA (Figure 3(a)).

*Expressions of miR-9 and miR-338-3p in the Epididymal Adipose Tissue of Mice with CL316243 Treatment*. Next we investigated the expression of the UCP1-targeting miRNAs in epididymal adipose tissue with CL316243 treatment. Compared with control group, as shown in Figure 3(b), the expression of miR-9 and miR-338-3p was significantly restrained in epididymal adipose tissue with CL316243 treatment (*P* < 0.05), and miR-let7g (not UCP1-targeting miRNAs, as negative control) expression had no statistical differences (*P* > 0.05).

## 4. Discussion

This study showed that there were more beige cells in epididymal adipose tissue with *β*3-adrenergic agonist (CL316243) treatment, labeled by significantly raised expression of brown adipose specific gene UCP1 and the appearance of lots of beige cells in HE sections. These results were consistent with previous researches on the browning effects of CL316243 in epididymal adipose tissue of animals [34–36]. The present study highlighted the potential involvement of miRNAs in the regulation of UCP1 expression on activating *β*3-adrenoceptor.

The BAT specific genes DIO2, CIDEA, and CPT1B mRNA were also induced in response to selective *β*3 agonist. Interestingly, UCP1 mRNA expression level was restrained in brown adipose tissue in CL316243 group although there was no significant difference. We estimated that the browning of epididymal adipose tissue may increase thermogenesis, so interscapular brown adipose tissue restrained its ability of generating heat to keep balance of body temperature.

Earlier studies show that enhanced expression of UCP1 in WAT of mice could reduce obesity [12, 37, 38]. Furthermore, the distribution of white adipose tissue (WAT) affects metabolic risk greatly. Increased volume of visceral adipose tissue is associated with a higher risk of metabolic disease [2]. Interestingly, these beige cells play a more important role than the interscapular BAT in mice losing weight following *β*-adrenergic or cold stimulation [39]. So browning visceral adipose tissue (e.g., epididymal adipose tissue) may be more effective to treat metabolic disease.

The exact mechanism involved in the appearance of beige cells in epididymal adipose tissue with a *β*3-adrenergic agonist is still not fully elucidated. As previously showed, several miRNAs have been demonstrated to be involved in activating of BAT or the process of browning of WAT. However, whether more miRNAs are involved in the process of WAT browning is still unknown. miRNAs bind to complementary target sites and lead to repression of translation or degradation of the target transcript [40]. Here we showed that miR-9 and miR-338-3p which possibly targeted UCP1 were dramatically decreased with CL316243 treatment. These results seemed to strictly correlate with an increase in UCP1 mRNA, confirming the hypothesis that CL316243 could reduce miR-9 and miR-338-3p expression leading to reducing the degradation of UCP1 messenger RNA. We proposed that *β*3-adrenergic stimulations might restrain expression of miR-9 and miR-338-3p in the epididymal adipose tissue in mice.

Here our study showed that prediction of miRNAs according to the miRNA database may be an alternative way to identify novel miRNAs involved in the process of WAT browning. We focused on the UCP1-targeting miRNAs, which may indicate the process of browning WAT clearly and lay a good foundation for the prevention and treatment of obesity. Clearly, we need to identify whether miR-9 and miR-338-3p are the potential candidates that target UCP1. miRNAs by luciferase reporter assays and qRT-PCR identification are ongoing but beyond the scope of this paper.

Taken together, our findings suggest that potential UCP1-targeting miR-9 and miR-338-3p may be involved in the process of the browning of epididymal adipose tissue through posttranscriptional suppression of UCP1 gene expression.

## Figures and Tables

**Table 1:** Primer sequences for PCR analysis.

Gene/microRNA	Forward primer (5′-3′)	Reverse Primer (5′-3′)
UCP1	GGCAAAAACAGAAGGATTGC	TAAGCCGGCTGAGATCTTGT
PGC-1*α*	ACAGCTTTCTGGGTGGATTG	TGAGGACCGCTAGCAAGTTT
C/EBP*β*	TGACGCAACACACGTGTAACTG	AACAACCCCGCAGGAACAT
PRDM16	GAAGTCACAGGAGGACACGG	CTCGCTCCTCAACACACCTC
PPAR*γ*2	TCGCTGATGCACTGCCTATG	GAGAGGTCCACAGAGCTGATT
CIDEA	TGCTCTTCTGTATCGCCCAGT	GCCGTGTTAAGGAATCTGCTG
DIO2	TGTCTGGAACAGCTTCCTCC	CCATCAGCGGTCTTCTCCG
CPT1B	CCAGACCCATACACCGACAG	GTCTCAGAGCCTCCCGATA
Cyclophilin A	GCATACAGGTCCTGGCATCT	ATCCAGCCATTCAGTCTTGG
miR-9	GCTCTTTGGTTATCTAGCTGTATGA	
miR-338-3P	GCTCCAGCATCAGTGATTTTGTTG	
miR-let7g	GGCCGTGAGGTAGTAGTTTGTACAGTT	
U6	TACGATCGCTTCGGCAGCACATA	

## Abbreviations

BAT: Brown adipose tissue
WAT: White adipose tissue
miRNAs: MicroRNAs
UCP1: Uncoupling protein 1
T3: Thyroxine.
